# Early Surgery for Acute Cholecystitis in the Elderly: Getting it Right the First Time

**DOI:** 10.1007/s00268-023-06992-9

**Published:** 2023-03-29

**Authors:** Ayham Alshbib, Kjetil Søreide

**Affiliations:** 1 Department of Gastrointestinal Surgery, Stavanger University Hospital, Stavanger, Norway; 2 SAFER Surgery, Surgical Research Group, Stavanger University Hospital, Stavanger, Norway; 3 Department of Clinical Medicine, University of Bergen, Bergen, Norway

## Introduction

Acute cholecystitis is one of the most common surgical emergency presentations globally. In the USA alone, over 300.000 patients per year present to the emergency department with acute cholecystitis. The condition is more likely to occur with increasing age. In the past, a ‘three-day window’ was referred to as the safety zone in which one could perform an emergency cholecystectomy. Recent guidelines suggest that this time window can be extended up to 7 days and even within 10 days from the onset of symptoms [1]. Of note, the most recent World Society of Emergency Surgery (WSES) guideline states that: “*Early laparoscopic cholecystectomy (ELC) should be the standard of care whenever possible, even in subgroups of patients who are considered fragile, such as the elderly; those with cardiac disease, renal disease, and cirrhosis; or those who are generally at high risk for surgery*” [1].

However, compliance in clinical practice may not follow the guideline suggestions. Variation management of acute cholecystitis is considerable. In one study from the United Kingdom (UK), the predicted probability of getting an emergency cholecystectomy during admission ranged from almost everyone (98%) to almost none (2%) [2]. In another study in the UK National Health Service (NHS), 50% did not undergo cholecystectomy during the same admission [3]. This documented variation in management likely reflects a variation in the provision of care, but also in variation in access to emergency theaters and in variation in dedicated teams (not only surgeons) for emergency general surgery in hospitals. Hence, it is most likely that we underserve, undertreat, and underdeliver sufficient care for a large proportion of patients admitted with acute cholecystitis. We can only reflect on the provision of care in our own country in Norway, where even the universal health care system does not provide timely and efficient care for all patients in large, populated areas due to lack of access to emergency theaters and due to competing emergencies. Another argument often used is that elderly and frail patients do not tolerate surgery as well, and hence, surgery is deferred or not done altogether. Indeed, frailty is associated with higher morbidity and mortality after laparoscopic cholecystectomy for acute cholecystitis [4]. However, the elderly may also benefit from timely surgical care.

It is thus somewhat reassuring to see the results provided in this issue of the Journal in a study from colleagues “down under” [5]. In a population-based registry cohort of over 47,000 patients from New South Wales, they found that a very high rate (85%) of cholecystectomy was performed in elderly patients with acute cholecystitis within 7 days of admission. Early surgery was associated with several favorable outcomes, such as shorter overall stay, fewer readmissions, fewer conversions to open surgery and a lower bile duct injury rate. Delayed surgery was associated with increasing age, male sex, higher comorbidity level, public hospitals, and low-volume hospitals. Also, patients who underwent early surgery were more likely to live in a major city.

Uncertainty persist towards what constitutes the most optimal surgical care and timing in the elderly with acute cholecystitis [6]. Surgery for elderly patients is increasing for several reasons: an increasing life expectancy; improved health in the elderly; better medical and surgical management of comorbidities; and treatment offered at a higher age and even with lower risk than in the past. However, with increasing age comes an increase in the considerations required for surgery. In acute cholecystitis, a single rule that fits “all patients” cannot be applied, and there is no validated way of stratifying risk in elderly patients. As such, conservative management with antibiotics for acute cholecystitis may be the right choice in some elderly patients, but it may also pose a major challenge due to interaction with other medications, presence of comorbidities (e.g., diabetes or kidney disease), and the need for prolonged care. Surgery as the first choice, when possible, is thus associated with better outcomes in terms of shorter hospital stays and fewer overall complications.

The mortality rate of elderly patients is lower in those who have a cholecystectomy during the same admission compared to those managed conservatively. This may be due to a selection bias, whereby the sickest and most frail are not selected for surgery, as they may have a recognized high risk of death [4]. Acute cholecystitis may be the tipping point for some elderly, frail patients, and conservative measures, even palliation, may be the most appropriate management in some. In others, conservative means by antibiotics may be entertained and if medical therapy fails, a percutaneous cholecystostomy could be considered. In some, percutaneous drainage can be performed as an intent to bridge to delayed cholecystectomy in a “cool” phase with a more moderate risk profile and more suitable for elective surgery. In some, the decision to delay and defer surgery is appropriate––and may be final in some. However, many patients initially treated with a percutaneous drain never returned to have completed cholecystectomy. The reason for delayed surgery (or no surgery) is manyfold and not completely understood. However, the study in this issue suggests that if a hospital did > 3 acute cholecystectomies per week, this was associated with higher rates of early surgery. Most likely, this reflects the given hospital system’s preparedness to deal with acute gallbladders altogether. As noted, “Acute Surgical Units” have formed across Australia in the past decades––this work may indicate their effectiveness in use.

Despite the favorable results obtained, the study did demonstrate that delayed surgery increased with age, from 12.8% in the 50–64 years group to 16% in the 65–79 years group, and for every fifth (22%) in the 80–84 years group and every fourth (24%) in the >  85 years group. Hence, while we should strive for optimal surgical care for the elderly with acute cholecystitis, we should also strive to understand for whom surgery is not a viable first choice. However, as demonstrated by the data from New South Wales in Australia, the secret of optimal outcomes in the elderly population with acute cholecystitis lies in getting it right the first time.
